# Heart Rate Variability: A Personal Journey

**DOI:** 10.1007/s10484-022-09559-x

**Published:** 2022-09-22

**Authors:** Stephen W. Porges

**Affiliations:** 1 Traumatic Stress Research Consortium, Kinsey Institute, Indiana University Bloomington, Bloomington, USA; 2 University of North Carolina at Chapel Hill, Chapel Hill, USA

**Keywords:** Heart rate variability, Autonomic nervous system, Polyvagal Theory, Vagus

## Abstract

Heart rate variabfility (HRV) has been a focal point throughout my academic history. To put into perspective, I have published studies spanning seven decades focusing on HRV (1969–2022). My interest in HRV started early in graduate school and continues to be an important portal informing my theoretical perspective. The current paper tracks some of this history, which started as an empirical observation and moved through several scientific stages including development of quantitative methods and investigations of neural mechanisms. Along this journey a variety of hypotheses were tested including the relative sensitivity of HRV metrics to neural mechanisms, psychological processes, and medical diagnoses. In addition, the research led to the identification of portal of intervention that have become strategies to optimize mental and physical health. These apparent disparate programs of inquiry have been tightly merged as the Polyvagal Theory evolved. In the sections below, I have shared my personal journey through these stages of scientific inquiry and my attempts to integrate the new knowledge in an expansive theoretical model.

## Personal History

In the fall of 1966, I arrived at Michigan State University, as an entering graduate student in a PhD program in psychology. At that time Psychophysiology was emerging as a new discipline bridging psychology and physiology. It was a discipline with a scant literature. Few books and articles on the topic had been published. Only two years earlier, a new journal, Psychophysiology, was founded to provide a home for peer-reviewed research in this area. Previously psychophysiological research had been buried in the Journal of Experimental Psychology and Psychosomatic Medicine. At the time, I did not appreciate my role as a pioneer, although the founding of the Society for Psychophysiological Research had only occurred six years earlier. Before we focus on the state of the emerging discipline of psychophysiology and my role in bringing interest in HRV research, it is important to see psychology and graduate training in psychology from a historical perspective.

On a personal level, my youth may have influenced my understanding of the history of psychology as an academic discipline. A student entering graduate school approaches novelty in ways that are like young children who are born into a pre-existing family structure or are confronted with cultural institutions and expectations when they enter school and community. The child assumes that the context is stable and initially sets out to learn the rules without attempting to change them. Similarly, our intellectual perspective of a discipline is distorted by academic experience and young initiates in a discipline see their field differently than the founders. Although it was clear to me that Psychophysiology was new and an innovative approach to investigate historic brain-body or even mind–body questions, I had not fully appreciated that the entire field of psychology was a youthful discipline that only emerged as an independent discipline during the academic careers of several of my mentors. At Michigan State University, psychology did not become an independent department until 1946, when it separated from the Department of Philosophy and Psychology. A few of my professors started their academic careers in a department dominated by philosophers and not scientists.

As an entering graduate student, I assumed that psychology as a discipline, like biology, physics, and chemistry, was permanently etched into the structure of academics. I did not have a sense that psychology departments were relatively recent additions in many colleges and universities. Nor did I assume that academic disciplines would need to fluidly adapt to a rapidly changing scientific literature. Perhaps, when you are 21 years old, you are less interested in the past and events that occurred 20–50 years before appearing less relevant. Perhaps, this was due my own experiences of being born at the end of World War II, a period of rapid change and forward thinking that emphasized the future and did not dwell on the past. As our culture becomes more trauma informed, we start to understand and appreciate the adaptive strategies of culture as it dynamically adjusted to the consequences of the collective trauma and devastation of war and the profound loss of agency felt by many during the depression. Similar to personal trauma, reflection or rumination on societal trauma can numb our nervous system and interfere with our innate need to be social and our intellectual passions to create and discover and even a desire to uncover our roots.

Through the lens of our trauma-informed world, our optimistic dreams of equality and the opportunities to express our intellectual and ethical potential were, in part, an adaptive form of a societal dissociation. In any case, amid civil unrest and protests driven by the Vietnam war and civil rights movement, I was part of an optimistic subculture that was being driven by expansive opportunities for higher education and the rapid growth and accessibility of graduate programs. At the time I entered graduate school, a PhD from a respected program was usually sufficient to be hired without post-doctoral training or additional teaching experience as an Assistant Professor in a strong PhD granting department.

This optimism was evident in my incoming classmates. Many came from uneducated families and were the first of their family to attend college. They shared a commitment to education, which several frequently verbalized as a firm desire to level the playing field of opportunities through education. Let’s not forget, that in the mid-1960s misogyny was an accepted feature of graduate education. I recall the orientation meeting of all the incoming graduate students, which started with a faculty member looking at the class and inquiring why so many women (about 50%) were in the room. He continued to suggest that most would not complete degrees. All these cultural features were familiar to me as a 21-year-old.

When I elected to go to graduate school, I was enamored by the expansive questions that psychology encompassed. I was especially interested in the internal conflict between intentional behaviors and emotional state. Later, I would focus on the neurophysiological platform for emotion, autonomic state. My research would naturally flow from developing tools to monitor autonomic state (i.e., HRV), to link autonomic state with mental and behavioral processes, to identify mental and physical health vulnerabilities, and develop interventions that would optimize autonomic regulation.

However, when I arrived as an entering student in the broad area of experimental psychology, the available research that professors were studying did not seem to match my interests. At that time the research conducted by the Michigan State faculty in experimental psychology focused on verbal learning, operant learning, classical perception, physiological psychology, and child development. However, within experimental psychology, there were a couple young faculty, who were interested in the emerging area of psychophysiology, an area that would enable empirical research of some of the historical mind–body questions linking feelings to performance by measuring physiological variables. By the fall of 1967, when I entered my second year of training, I had found a good match in being mentored by David Raskin. David was a young Associate Professor in his early 30s. He had been trained in physiological monitoring at UCLA by an established scientist, Irving Maltzman. His area focused on the bridge between learning and autonomic regulation that was a central topic in the scientists who followed Pavlov. These scientists were interested in classical conditioning and the unconditioned reactions to stimuli. In retrospect, the orienting and defensive responses may have been the intellectual trigger for the construct of neuroception in Polyvagal Theory (Morton et al., 2022; Porges, 2003, 2004), which functions as a reflexive detection of cues as being safe or threatening.

**Fig. 1:**
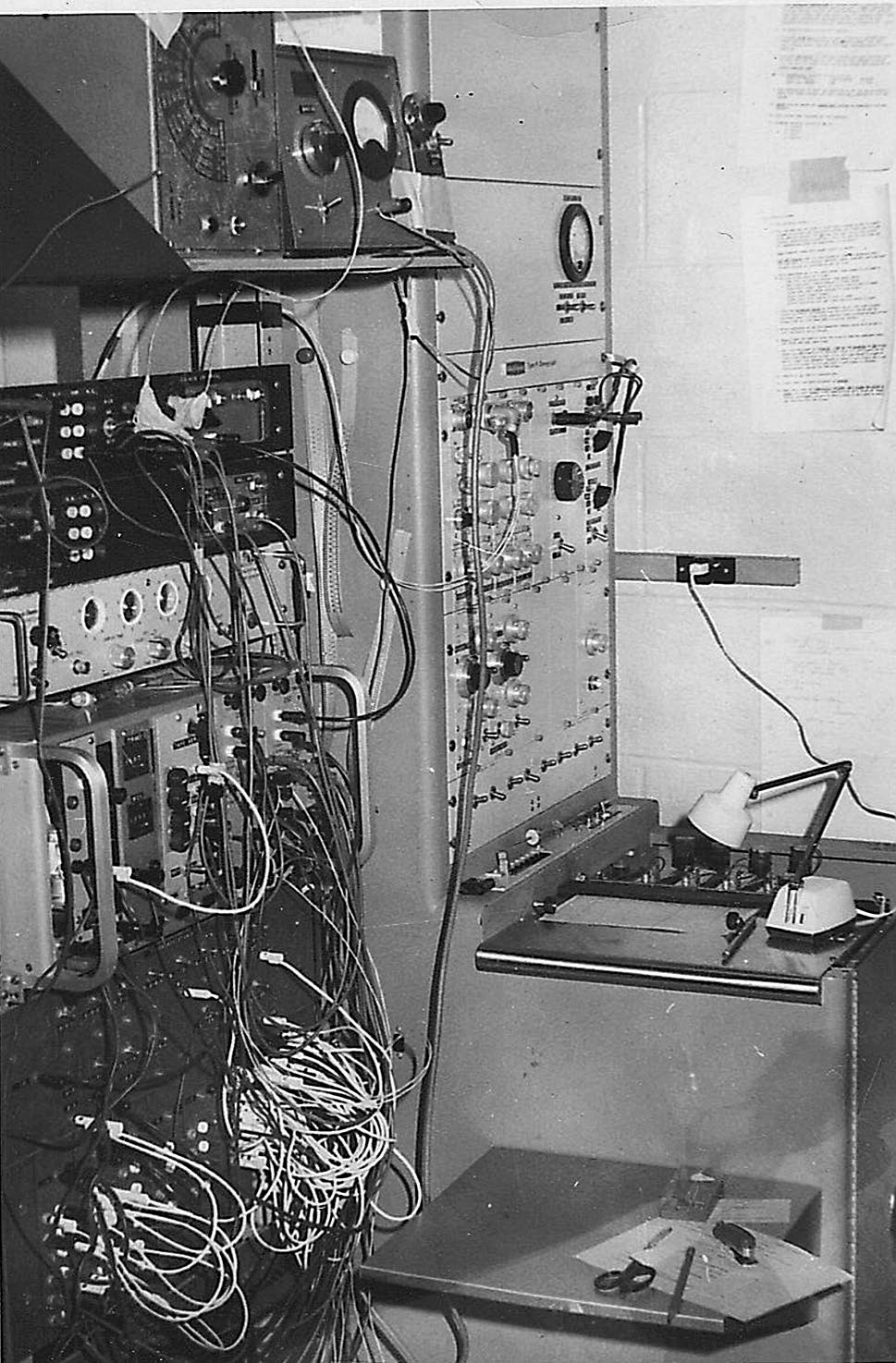
David Raskin’s laboratory circa 1967.

I became David’s mentee and teaching assistant for an undergraduate laboratory course in psychophysiology and he supervised my master’s research (Porges & Raskin, 1969). David had a great influence on my research. He emphasized an empirical and quantitative perspective that effectively balanced my expansive thinking. He provided an opportunity for me to develop skills in monitoring and quantifying autonomic activity. David was a cautious scientist and thought like an engineer. He triggered my interests in designing and fabricating equipment. These interests continue as I have been awarded several patents related to monitoring and regulating autonomic function. A picture of the laboratory equipment used for my Masters’ research is illustrated in Fig. 1. The equipment on the left of the picture is the relay rack that was used to control the stimulus presentation of sounds and lights. This equipment was constructed by the technician in the psychology department from parts scavenged from a government salvage facility available to universities and other public institutions. On the right is a Beckman Dynograph, a physiological monitor with ink pens scrolling out in real time beat-to-beat heart rate changes from a cardiotachometer and electrodermal changes.

It was during the pilot phase of research for my master’s thesis that I serendipitously observed systematic changes in heart rate variability during sustained attention. I was interested in identifying mental effort and intentionality from physiological signals. In a way, I was curious about what we could learn about human behavior from our bodily reactions without requiring a verbal response. As I watched the heart rate pattern being displayed on the polygraph paper, I noted that for several of the subjects the beat-to-beat heart rate pattern stabilized during the attention tasks and then returned to a baseline pattern that appeared to be systematically rhythmic at a frequency similar to spontaneous breathing. This was a new phenomenon, there was no literature of a respiratory pattern in beat-to-beat heart rate being sensitive to psychological demands linked to mental effort such as sustained attention. I immediately ask David what he thought. This started a discussion about potential mediating effects of breathing. Was the effect in heart rate being driven by a shift in breathing patterns or was there unique information in the heart rate pattern? We couldn’t answer this question since we did not have a sensor to measure respiration. To solve this problem, we temporarily pause the experiment and David purchased a respiration sensor for the Dynograph. After a few weeks I returned to collecting data.

**Fig. 2:**
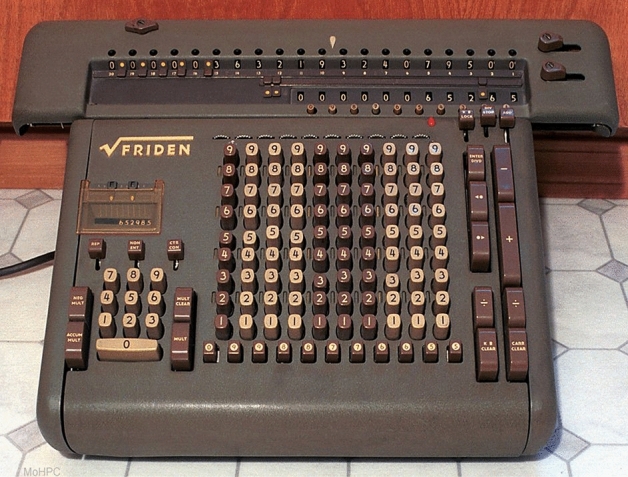
Friden mechanical calculator circa 1966.

**Fig. 3:**
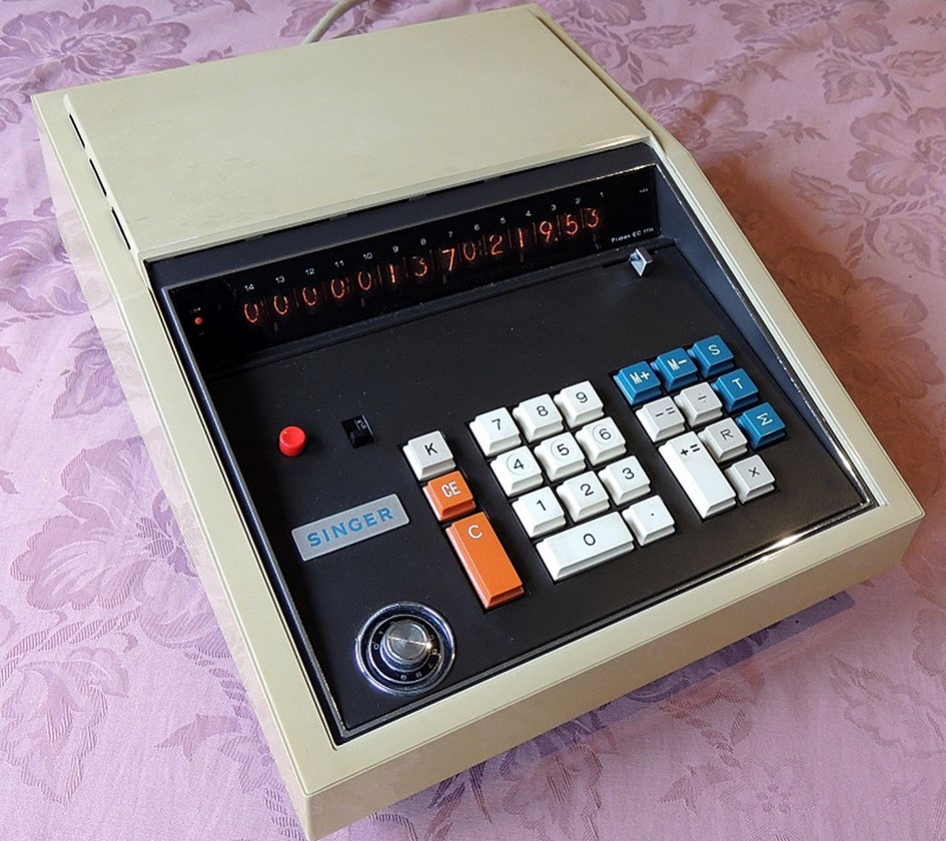
Early electronic calculator circa 1969.

During the 1960s computers were not available in individual psychology laboratories. In fact, this was a time when the major computational tools in psychology departments were mechanical calculators such as the Friden calculator (see Fig. 2). As I was completing graduate school, this was replaced by a digital desktop calculator (see Fig. 3). Most graduate students tediously conducted their analyses using these archaic devices. However, those of us collecting large arrays of beat-to-beat data had several additional challenges. First, we had to quantifying the beat-to-beat tracings from the Dynograph (see Fig. 1). This was done with a millimeter ruler. Thanks to the innovation of the cardiotachometer heart rate was a calibrated deflection on the paper. Prior to the cardiotachometer, heart rate was derived by measuring the interval between R-waves on the paper. However, with this technique precision was dependent on paper speed. Thus, more precision required more paper and paper for the Dynogtraph was very expensive. Heart rate was sampled second-by- second within the experimental conditions. Similarly, respiration was analyzed by counting the frequency of inspirations and mean amplitude of completed inspirations within each experimental condition. As each value was scored, it was entered into a notebook. With this notebook, I would go to the computer center and punch the ‘IBM’ cards. Then I carried 100s of data cards to the mainframe computer (CDC 3600/CDC 6400) to conduct the analyses of variance. Prior to the analyses, the data were subjected to a two-pass verification process during which the data were re-entered and compared with the original card. If there were no differences, a verification notch was punched on the right edge of the card and the data could be submitted for analyses and placed in a queue. Often the delay between submitting data and analyses would be several days. Once analyzed a large printout was placed in a bin that the user could retrieve. If a mistake was made on the format card, which instructed the computer what columns had specific data, a few days later you would pick up a very slim printout with the word ERROR prominently displayed. This type of error could cause major delays in completing the work and even scheduling thesis defenses.

Now back to the findings from my first HRV study. Based on our intuitive scoring of the data, we calculated the variance of the second-by-second heart rate. First, the results documented that the traditional variables of heart rate, respiration amplitude, and respiration frequency exhibited a systematic reaction during attention and were not selectively sensitive to variations in attentional demands. Basically, during the attention tasks breathing became more rapid and shallow, while mean heart rate increased. Over repeated trials the task effect on heart rate dissipated. Interestingly, the HRV data uniquely documented differential task demands linking suppression of HRV with sustained attention (Porges & Raskin, 1969). This finding documenting that mental effort resulted in a reduction of HRV was followed by my dissertation focused on linking HRV to reaction time performance and heart rate reactivity (Porges, 1972).

As I was finishing my Masters’ research, David moved from Michigan State University to the University of Utah. When he left, serendipitously I started to work with Hiram Fitzgerald, a young assistant professor in his late 20s. Hi was a developmental psychologist with an interest in infancy and early development. It was through Hi’s benevolent mentorship that I was welcomed into the world of developmental psychology, which has greatly enriched my theoretical perspective. His influence led to my interest in neonatal and prenatal autonomic regulation. The dissertation uncovered another important finding in my HRV journey. It documented that both the HRV level prior to the task and the suppression of HRV during the predicted reaction time. These findings set the stage for future research in individual differences and mental effort. The findings also influenced my research agenda by directing me into the interdependent challenges of understanding the neural mechanisms mediating HRV, developing methodologies to quantify HRV, and studying the maturation of the neural mechanisms.

The academic marketplace was far different from what it is now. When I finished my PhD at the age of 25 there was an expectation that I would have an academic job. This expectation was not atypical, and during my fourth year in graduate school I was offered attractive positions. I accepted a position at West Virginia University with an expectation that I would create a developmental psychophysiology research program. WVU, like other growing departments, had an optimistic perspective and was expanding, hiring both a young cohort and also seasoned established full professors. The department had a plan to become a visible program with a strength focusing on life-span developmental psychology. This seemed like a perfect place to start my academic career. At WVU I was given access to research space in the University hospital’s newborn nursery, where I studied the heart rate patterns of newborns to visual and auditory stimuli and conducted a study on temporal conditioning. After two years, I left WVU to move to the University of Illinois at Urbana-Champaign, where I joined the Department of Psychology. Over the next 50 years I have been in several universities and affiliated with several academic programs, however my research has continued to be focused on the autonomic nervous system and the important information that can be obtained through the quantification of HRV. Rather than elaborating on how my research bridged numerous disciplines and applications, the remaining parts of the paper provide a history of the antecedent science from which my work on the Polyvagal Theory evolved.

## Where Did HRV Start: A Dependence on Technology^1^

Although the research questions in psychophysiology are dependent on theory linking physiological responses to psychological and behavioral processes, these brain-body questions cannot be empirically investigated without devices that can measure bio-electrical potentials driven by the heart, brain, muscles, and skin as well as the physical changes associated with thoracic and abdominal activity during breathing. The history of the origin of these devices and their role in the study of HRV is described in this section. Astute students of psychophysiology will note that segments of this section reflect my contributions to a previously published guidelines paper (Berntson et al., 1997).

The ability to monitor, to conceptualize, and to interpret HRV is dependent upon both the technologies for observing the beating of the heart and the methodologies for quantifying heart rate parameters. These technologies and methodologies metaphorically provide windows of observation, resulting in either clear or blurred representations. Most of the research investigating HRV has occurred during the past 50 years. Clinical interpretations and applications have even a shorter history. However, before identifying the historical turning points in the study of HRV, I will provide a short sojourn into the prehistory of HRV.

Long before the invention of the electrocardiograph and the more recent emergence of the construct of HRV, physicians realized the importance of the heart and the rhythms of the beating heart. For several hundred years, physicians used auscultation to determine heart tones and heartbeat rhythms. Although the focus of auscultation is on listening to the closure of the heart valves, for centuries physicians have noted beat-to-beat rhythm shifts associated with aging, illness, and psychological states. The study of these rhythms became a central component of various medical diagnostic systems developed in India and China.

The scientific investigation of beat-to-beat heart rate rhythms was delayed until specific technological advances enabled accurate and reliable quantification of the electrical activity of the heart. This technology progressed through the development of four devices: the galvanometer, the kymograph, the ink writing polygraph, and the ECG.

The work of Galvani and later that of Alessandro Volta, the discoverer of electrical current, led to the development of the galvanometer. The galvanometer is a device that measures the amount of electrical current, by converting electrical energy into the physical displacement of a coil, which in turns moves a pointer. Through the application of Ohm's law, the galvanometer could be calibrated to accurately measure changes in voltage, even voltages in the range of bio-potentials generated by the heart. Ludwig (1847) invented the smoked kymograph that allowed mechanical activity to be recorded on a smoked drum. MacKenzie (1910), after toiling with the cumbersome kymograph, developed an ink-writing polygraph. In the 1890s Einthoven (see Erschler, 1988) integrated the galvanometer with photography to produce accurate and continuous “tracings” of the electrical activity of the heart.

Once the electrocardiograph was developed, it was possible to monitor normal and abnormal electrical conduction through the myocardia as well as to evaluate beat-to-beat changes in the heart rate pattern. Modern electrocardiographs are elaborations of the early Einthoven concept with the addition of paper recordings or computerized outputs.

The origins of the “scientific” study of HRV predate the ECG. The first documented observation of HRV may be credited to Hales (1733), who observed a respiratory pattern in the blood pressure and pulse in a horse. However, one might argue that the scientific investigation of HRV required quantification, and started with Ludwig's report that, with the kymograph, he was able to observe a regular quickening of pulse rate with inspiration and a slowing with exhalation in the dog (Ludwig, 1847). Ludwig’s observation may have been the first documented report of respiratory sinus arrhythmia (RSA), the rhythmic variations in the heart rate at the frequency of breathing. RSA, which is mediated by vagal cardioinhibitory pathways (see below) has been the central focus of my psychophysiological research as well as a target variable for biofeedback paradigms.

In 1865 Traube (cited in Anrep et al., 1936a) speculated on the neurophysiological mechanisms mediating RSA and proposed that the brainstem nuclei controlling heart rate might be phase dependent and influence heart rate by direct neural pathways from the medullary respiratory centers. Interestingly, more recent foundational neuroanatomical and neurophysiological data support a contemporary version of this model. Specifically, Richter and Spyer (1990) proposed a common cardiopulmonary oscillator that was dependent on the interneuronal communication between brainstem structures involved in the vagal regulation of the heart and the bronchi (i.e., nucleus ambiguus and nucleus tractus solitarius) driving similar oscillations in heart rate (i.e., RSA) and the bronchi. The phase relationship between oscillations in heart rate and bronchi was proposed in Polyvagal Theory (Porges, 1995) and more recently documented (Giardino et al., 2003) to optimize diffusion of oxygen. Basically, the oscillatory rise in heart rate produces a transitory increase in blood pressure that is phase optimized with bronchial oscillations to literally push oxygen into the blood during inhalation.

In 1871 Hering (see Anrep et al., 1936a) proposed an alternative, but not necessarily contradictive explanation. Hering proposed that reflex modulation of the cardio-regulatory centers by pulmonary afferent feedback may be the physiological mechanisms underlying RSA. In addition, he reported that the systematic pattern of heart rate changes associated with breathing diminished with advancing age, a point that we have confirmed (see Byrne et al., 1996). But perhaps most insightful and relevant to contemporary psychophysiology and RSA focused biofeedback, was Hering’s observation (1910) that he could identify cardioinhibitory fibers traveling from the brainstem down to the heart, through the vagus. As he so succinctly put it: *“It is known with breathing that a demonstrable lowering of heart rate… is indicative of the function of the vagi.”* Hering’s observation that breathing lowers our heart rate, and that the vagus is behind this slowdown, was hugely foundational for the development of the Polyvagal Theory. It also gave psychophysiologists and physiologists a neurophysiological basis to develop a non-invasive method for measuring vagal activity using the RSA component of HRV. In addition, it gave biofeedback researchers a validated neural portal to target and monitor. With the ability to easily measure vagal activity various hypotheses derived from the Polyvagal Theory could be objectively tested. This later point is critical in understanding the focus on quantitative methods to refine estimates of vagal activity in my research.

The history of the study of HRV is dependent upon the ability and strategy to quantify beat-to-beat activity. Accurate timing of beat-to-beat variability was dependent upon the detection of features of ECG (e.g., peak of the R-wave), and thus the era of scientific investigation of HRV was dependent upon the availability of the electrocardiograph for physiological and clinical research. As described above, specific electrophysiological devices were required to provide accurate measures of cardiac activity prior to the development of the research area. Research has been aided by techniques to detect and accurately time the onset of sequential heart beats. Engineers developed electrical circuits to identify the peak of R-waves and timers that evolved into tachometers. With the advent of laboratory computers, timing became more precise and accurate and computer algorithms were able to detect R-waves and other components of the ECG.

Although not exhaustive, several historical studies highlight the emergence of HRV as a physiologically meaningful measure. Most of these early clinical and physiological studies have focused on RSA. In fact, in early research there is little distinction between a global concept of sinus arrhythmia and the more specific rhythmicity of RSA. However, from my perspective the descriptive statistics frequently used to quantify HRV are agnostic to neural mechanisms and have frequently diverted researchers from neural mechanism, while RSA is implicitly a metric dependent on specific neural pathways embedded in the vagus (see section below on quantification).

From several scientific sources, references to RSA were made in the early 1900s. Even in the early psychology textbooks, there is mention of the phenomenon that later became known as RSA. Wundt (Wundt & Judd, 1902) stated that “… the movements of the lungs: their inflation accelerates, their collapse reduces the frequency of heartbeat. The respiratory movements are therefore regularly accompanied by fluctuations of the pulse, whose rapidity increases in inspiration and decreases in expiration.”

Research on HRV moved, initially, in two directions. First, there was a dominant trend towards understanding the physiological mechanisms mediating HRV. Second, clinical medicine identified specific relationships between HRV and clinical status. These two directions co-existed prior to the emergence of psychophysiology. However, in the 1960’s with the availability of polygraphs in academic laboratories, a third trend appeared when psychophysiologists started to investigate the relationship between psychological processes and HRV. This trend also included the application of learning and conditioning strategies to modify HRV, which lead to the emergence of biofeedback as a discipline focusing on clinical treatment strategies. As the third trend expanded research, HRV became an accepted variable capable of indexing resilience and vulnerability in medical and mental health. With this interest, researchers became intrigued with the possibility of enhancing HRV through biofeedback as a clinical technology to optimize health. This clinical and applied interest in modifying autonomic regulation led to the establishment in 1969 of the Biofeedback Research Society, which evolved into the Association for Applied Psychophysiology and Biofeedback.

Bainbridge (1920) provides an early example of physiological research on HRV when he attempted to explain RSA in terms of alterations in baroreceptor and volume receptor responses to changes in blood flow caused by changes in thoracic pressure associated with respiration. Anrep et al. (1936a, 1936b) pursued alternative explanations of the physiological mechanisms mediating RSA. They published, what appears to be, the first extensive study of RSA. Anrep and his colleagues investigated the influence of several physiological parameters on RSA, including: the influence of respiratory rate and amplitude, blood gas concentrations, and efferent cardio-regulatory neural pathways.

Eppinger and Hess (1915) published a monograph in the Journal of Nervous and Mental Disease called ‘vagotonia.’ The publication illustrated an interest in autonomic function within the emerging area of psychiatry. The focus of their monograph was to emphasize that an apparent ‘hyperactivated’ vagus was associated with mental disorders. Their monograph provided a starting point for the clinical trend. They stated that “… clinical facts, such as respiratory arrhythmia, habitual bradycardia, etc., have furnished the means of drawing our attention to the variations in the tonus of the vagal system in man.” Although Eppinger and Hess were interested in clinical medicine, their case studies described a relation between a clinical problem in the regulation of autonomic function that did not have, with the available technology, a morphological correlate. Their observations are relevant to contemporary psychophysiological investigation of HRV and the emerging discipline of functional medicine for several reasons including: (1) they alerted us to the importance of the autonomic nervous system in mediating atypical physiological responses; (2) they related individual differences in physiology to individual differences in psychiatric pathology (i.e., neuroses); (3) they recognized the pharmacological sensitivity of the vagus to cholinergic agents, thereby potentially identifying pharmacological treatments; and (4) they brought to the attention of the medical community the commonalty of the vagal innervation of various peripheral organs and thus a possible common explanation for several clinical disorders.

Clinical research interests were rekindled in cardiology by the early research of Wolf (1967) and in obstetrics and gynecology by Hon (see Hon & Lee, 1963). Both Hon and Wolf emphasized the relationship between HRV and nervous system status. Hon treating HRV as a global index of clinical viability of the fetus and neonate. Wolf, with his focus on the contribution of central nervous system factors to sudden cardiac death, viewed HRV as representing brain-vagal-heart communication. Wolf's research with a theoretical interest in brain–heart relations provides an important bridge between clinical research and psychophysiology.

## Quantification

Heart rate patterns are complicated and often idiosyncratic time series. Although we now know that the beat-to-beat pattern is continuously influenced by the changing neural influence from the brainstem to the heart, within psychophysiology the focus on the neural component was often neglected in publications and presentations. The procedures selected to quantify HRV are critical both in extracting physiologically meaningful components and in building a plausible neurophysiological model relating physiological activity to behavior and psychological processes.

There are two basic approaches to the quantification of HRV: (1) the use of descriptive statistics (i.e., range, standard deviation, variance, etc.) and (2) the modelling of the heart rate pattern to extract variance components defined by amplitude and frequency determined by known physiological mechanisms (e.g., RSA). While both approaches produce descriptive statistics, the descriptive approach is influenced by the duration of the data sampled, the mean level and the complex interactions of various neural influences on the overall HRV. In is important to note that the two approaches are not convergent with the prevalent distinction of time domain versus frequency domain. Time domain methods can be used to model periodic processes and, if the data are statistically stationary, any frequency domain method can be transformed into a time domain method (Brillinger, 1975). However, time domain representations may have advantages when data are not statistically stationary (Bohrer & Porges, 1982; Porges & Bohrer, 1990). For example, since psychophysiologists are interested in the changing level of variables such as RSA, the data would be nonstationary. For example, my work with Bob Bohrer, a mathematician resulted in an innovative time domain method to study the changing amplitude of periodic components, even in dynamic situations (e.g., Bohrer & Porges, 1982; Porges & Bohrer, 1990; Porges, 1986a, 1986b, 1985a, 1985b).

The early history of Psychophysiology focused on the use of descriptive statistics (e.g., Porges, 1972; Porges & Raskin, 1969). By the late 1970’s there was interest in a different, and perhaps more precise way of quantifying HRV via time series analyses. Time series analyses provided a method of modeling periodic components of the heart rate time series and in extracting periodic components that might have physiological and psychophysiological significance. With the support of my collaborator, Robert Bohrer, we organized a symposium and two workshops on the application of time series analyses of HRV that were presented to the membership of the Society for Psychophysiological Research. In 1978 at the Madison, Wisconsin meeting, we organized a preconference symposium on the potential applications of spectral and cross-spectral analyses for the quantification of HRV and respiratory-heart rate coupling. This was followed in 1983 when we conducted a full-day workshop on spectral analyses at the Asilomar meeting. In 1984 we conducted a second full day workshop on HRV at the Milwaukee meeting. These well attended workshops served to stimulate many investigators to apply spectral analyses and other time series methods to the study of HRV.

The measurement of HRV also illustrates a historical trend. In early clinical research HRV was quantified as a descriptive statistic of range or standard deviation. For example, during the past 50 years clinicians in obstetrics and neonatology (e.g., Hon & Lee, 1963), have defined HRV in terms of the standard deviation of beat-to-beat values over short durations (i.e., short term variability) or the range over longer periods (i.e., long term variability). In basic research areas newer methods were introduced to extract periodic components from the heart rate pattern. Chess et al. (1975) introduced spectral analyses to the measurement of HRV. Porges and colleagues (1980) introduced cross-spectral analysis as a method of evaluating the linkage between respiration and HRV in humans and proposed that the sum of the spectral densities in the heart rate spectrum associated with the respiratory rhythms was an accurate estimate of vagal tone (1985b). Akselrod et al. (1981) applied spectral analysis to dog heart rate and demonstrated that the respiratory rhythm in the heart rate spectrum was related to vagal tone. In addition, they identified two slower frequency bands, which they presumed were related to an interaction between vagal and sympathetic influences. Although commonly assumed, there is no reliable evidence that the slower frequencies or the ratio between the slower frequencies and RSA reflect sympathetic influences or can be used as an index of sympathovagal balance (see. Eckberg, 1997). In addition, research in my laboratory clearly documents that virtually all periodic variability is determined via cholinergic pathways assumed to travel through the vagus (Grippo et al., 2007; Porges, 2007). These observations do not preclude potential interactions with SNS, but the lower frequency HRV components are certainly not a direct index of the SNS.

In the early 1980s my laboratory introduced a time–frequency methodology (Porges & Bohrer, 1990; Porges, 1985a). This methodology has been documented to be significantly more sensitive to vagal influences than the traditional time and frequency domain method (see Lewis et al., 2012). Our time–frequency methodology, by enabling estimates of RSA during short epoch (e.g., 10–15 s), provided an opportunity to ask new questions. With short estimates the dynamic relationship between RSA and heart rate could be quantified. Functionally, the metric provides a measure of *vagal efficiency* operationally defined as the slope of the regression. The slope provides a metric that describes the ms change in heart period that would occur with a change of one log unit of RSA amplitude. The initial study documented that newborn sleep state could be reliable detected by this method (Porges et al., 1999). This finding has recently been replicated with high-risk preterm infants and expanded to index clinical course (Porges et al., 2019). Additional studies indicated that in response to alcohol vagal efficiency decreased in humans (Reed et al., 1999.). As we became more familiar with the metric, systematic challenges were used. In a clinical study using a posture challenge protocol, it was possible to detect a subset of patients in a pediatric gastroenterology clinic with joint hypermobility syndrome (Kolacz et al., 2021). In another study, an exercise bike challenge was used in a study with college students and vagal efficiency distinguished participants with and without a maltreatment history (see Dale et al., 2022a). On a clinical level, it appears that vagal efficiency may provide an objective metric related to the clinical features associated with a diagnosis of dysautonomia. Our current research is focusing on vagal efficiency as being an objective neurophysiological index that may be associated with a broad array of functional disorders.

## Dependence on Theory and Theory as Filter

Psychophysiology has been the at crossroads of different models and strategies for research. Unlike physiology with its interest in mechanism, or cardiology with its interest in clinical status, psychophysiology has often been driven by paradigms derived from psychology focusing on demonstrating that physiological parameters are correlated with psychological and behavioral states. The early history of the Society for Psychophysiological Research and the early issues of *Psychophysiology* echo this strategy with psychophysiologists expressing an aphysiological treatment of physiological parameters. Thus, although the measures of physiological activity were operationally defined and diligently quantified, the articles and presentations were virtually devoid of a reference to the neural mechanisms of the physiological response variables. In the early days of the Society psychophysiologists treated physiological parameters similar to overt behavior or subjective reports with the exception that to study physiological activity it was first necessary to transform the physiological activity into an observable tracing on the polygraph.

At the time I first presented the Polyvagal Theory, arousal theory was the prevalent theoretical perspective in psychophysiology. Although arousal theory had a long, influential history in science, it had a relatively simplistic underlying model. Basically, arousal theory emphasized that arousal was a linear construct indexing a dimension from low to high levels of activation that could be measured or inferred from observing behavior or physiology. The relationship between arousal and performance was often portrayed as an inverted U- shaped function in which optimal performance occurred within a midlevel range, while poor performance was observed at low and high levels of arousal. This relationship was known as the Yerkes- Dodson law (Yerkes & Dodson, 1908). Metaphorically, arousal represented the energy of the human nervous system. Arousal was easily understood, since when it was reflected behaviorally it could be quantified as greater activity and when reflected autonomically it could be observed as increases in sweating and heart rate.

Early psychophysiological research assumed that peripheral autonomic measures provided sensitive indicators of arousal. This view was based on a rudimentary understanding of the autonomic nervous system in which changes in electrodermal activity (e.g., sweating) and heart rate were assumed to be accurate indicators of sympathetic activity. As the activation arousal theory developed, a continuity between peripheral autonomic responses and central mechanisms was assumed (see Darrow et al., 1942), and sympathetic activity was assumed to parallel activation of the brain. According to this assumption, organs influenced by sympathetic efferent fibers, such as the sweat glands, blood vessels, or the heart, were potential indicators of limbic or cortical activity (Duffy, 1957; Lindsley, 1951; Malmo, 1959).

Although the specific pathways relating these various levels were never outlined and are still sketchy, electrodermal (e.g., GSR) and heart rate became the primary focus of research during the early history of the Society for Psychophysiological Research. This was due to their presumed sympathetic innervation and, in part, to their measurement availability. By default, this emphasis created a research environment that neglected several important factors: (a) parasympathetic (e.g., vagal) influences, (b) interactions between sympathetic and parasympathetic processes, (c) peripheral autonomic afferents, (d) central regulatory structures, (e) the adaptive and dynamic nature of the autonomic nervous system, and (f) phylogenetic and ontogenetic differences in structural organization and function.

In general, arousal theory was a top-down model that implicitly led to attempts to translate from psychological to physiological phenomena, a strategy that could be succinctly labeled as “psychophysiological parallelism.” As a research strategy, psychophysiological parallelism makes a strong assumption that it is possible to identify unique neurophysiological signatures of specific mental processes (e.g., feelings, emotions, thoughts, etc.).

In the 1960s psychophysiology emerged as an interdisciplinary science with historic roots embedded in an assumed psychophysiological parallelism, which was consistent with arousal theory having parallel outputs in brain, ANS, and behavior. Although Polyvagal Theory (Porges, 1995) emerged from traditional psychophysiology, it provided a theoretical demarcation from parallelism. In a sense, psychophysiological parallelism implicitly assumed that of the constructs employed in different domains were valid (e.g., subjective, observable, physiological) and focused on establishing correlations across domains that optimistically would lead to an objectively quantifiable physiological signature of the construct explored in the psychological domain. In contrast, Polyvagal Theory focuses on the interactive and integrative aspects of different levels of the nervous system.

Polyvagal theory emphasizes a hierarchical organization that mirrors phylogenetic shifts among vertebrates. The evolutionary changes are also reflected in maturational trends. Thus, what appears to be more complex and related to higher brain structures, such as language and sensitivities to another’s physiological state via intonation of voice and gesture, is reflecting the functional and structural changes mapped into the evolutionary history of vertebrates. Frequently missed with our cortico-centric and cognitive-centric orientation is the importance of lower brain mechanisms in managing our basic survival-oriented reactions. Although the less complex earlier evolved systems are often repurposed in mammals, they remain survival oriented and are efficiently available to support states of defense when survival is challenged.

Psychophysiological parallelism is functionally a scientific strategy that assumes an isomorphic representation of a process with the gradations being mapped with equivalent precision on all levels. An alternate and more parsimonious strategy would be to organize the nervous system into a hierarchical format in which neurophysiological processes related to basic biologically determined survival needs are required to be managed successfully before higher brain structures are functionally given access to be activated for problem solving, creativity, and even sociality. Polyvagal Theory postulates that our biology is hierarchically organized with the basic survival needs, such as managing homeostatic functions, residing in foundational brainstem structures and optimal access and utilization of higher neural circuits being dependent on success at the foundational level. Polyvagal Theory provides insights into what the hierarchy is and how it can be identified and potentially monitored. According to the theory, the hierarchy reflects the phylogenetic shifts in the nervous system (see Porges, 2021, 2022).

Against the backdrop of arousal theory, psychophysiological parallelism, a bias toward static metrics of autonomic function (e.g., resting heart rate, blood pressure), and a limited understanding of how the dynamic regulation of the autonomic nervous system could support or disrupt homeostasis, Polyvagal Theory emerged on an October morning in 1994 as the focus of my presidential address at the annual meeting of the Society for Psychophysiological Research in Atlanta, Georgia. The presentation was formalized into a manuscript and published in the society’s journal, *Psychophysiology* (Porges, 1995). At that time, my objective was to archive the extracted principles from my previous 25 years of research and to challenge my discipline to explore autonomic reactivity from a new perspective. Although several of the principles were novel, the general questions were familiar to psychophysiologists, who were vested in exploring the utility of monitoring heart rate patterns to gain additional information about mental and health-related processes.

Polyvagal Theory extracts from contemporary neuroanatomy, neurophysiology, evolutionary biology several basic uncontroversial conclusions that can be succinctly summarized: (1) mammals have two vagal pathways (i.e., supradiaphragmatic, subdiaphragmatic), (2) evolution and development provide insight into the changes in brainstem structures that enable mammals to be physiologically calm and socially interact. Functionally, mammals have neural attributes that efficiently, via rapidly responding cardioinhibitory fibers (e.g., myelinated ventral vagal pathways originating in nucleus ambiguus), calm autonomic state and promote social communication. The ventral vagal pathways also coordinate and repurpose circuits that evolved to support defense in socially relevant processes (see Porges, 1998, 2007), such as play (i.e., ventral vagal influences constrain sympathetic reactivity), and intimacy (i.e., ventral vagal influences constrain dorsal vagal reactivity).

Through the study of neural development and phylogeny, we can extract foundational principles and their underlying mechanisms through which the autonomic nervous system leads to feelings of safety and opportunities to co-regulate. Thus, feelings of safety may reflect a core fundamental process that has enabled humans to survive through the opportunistic features of trusting social engagements that have co-regulatory capacities to mitigate the metabolically costly defense reactions (see Porges, 2021, 2022). The table below highlights the theory’s emphasis on adaptive processes linking autonomic state to health and sociality (Table 1).

**Table 1:** Features of the polyvagal theory

1	Polyvagal theory focuses on the foundational survival oriented neural circuits located in the brainstem that regulate the autonomic nervous system and support homeostatic processes
2	Polyvagal theory emphasizes the hierarchical nature of the nervous system with foundational survival focused circuits functioning as a neural platform for higher brain structures involved in cognitive and emotional functions (i.e., disruption of foundational circuits compromises higher brain processes)
3	Autonomic state functions as an intervening variable biasing reactions to events and even pathogens
4	Consistent with the Jacksonian principle of dissolution (Jackson, 1884) autonomic state is regulated by three neural circuits (i.e., ventral vagus, sympathetic nervous system, dorsal vagus) that form a phylogenetically and maturationally ordered hierarchy in which newer circuits inhibit older circuits
5	Within the vagus nerve there are two neuroanatomically distinct vagal efferent pathways that originate in different areas of the brainstem (ventral, dorsal)
6	The dorsal vagus originates in the dorsal nucleus of the vagus and has unmyelinated vagal motor fibers that primarily regulate organs below the diaphragm (e.g., gut). The dorsal vagus has unmyelinated cardioinhibitory pathways that appear in most situations to be dormant but may become activated in life threat situations (e.g., hypoxia)
7	The ventral nucleus of the vagus (i.e., nucleus ambiguus) is the source of myelinated cardioinhibitory pathways projecting to the heart in mammals
8	The ventral vagal cardioinhibitory pathways have a respiratory rhythm that is observed as RSA with the amplitude of RSA reflecting the chronic cardioinhibitory influence of the vagus (i.e., cardiac vagal tone) on the heart
9	Neuroanatomically the special visceral efferent pathways within cranial nerves V, VII, IX, X, and XI with the ventral vagus form an integrated social engagement system that regulates the neural structures involved in ingestion at birth and social communication throughout the lifespan
10	The social engagement system by recruiting the calming circuits associated with the ventral vagus enable social interactions (i.e., co-regulation) to constrain the defensive circuits involved in fight/flight and shutdown (e.g., death feigning) to support play and intimacy
11	Through the process of neuroception (i.e., the nervous system’s detection of signals of risk without awareness) signals of threat reflexively shift autonomic state to optimize survival through mobilization and immobilization strategies, while signals of safety calm autonomic state and support the homeostatic processes of health, growth, and restoration
12	Feelings of safety reflect an autonomic state supporting homeostatic processes and form the foundational neural platform for sociality

Polyvagal Theory did not fit well within the constraints of arousal theory, although Polyvagal Theory could provide a neural explanation of arousal theory. Arousal theory fit an outdated, but still taught, model of the autonomic nervous system that interpreted arousal as a competition between the sympathetic and parasympathetic nervous systems reflected in autonomic balance. However, it did not provide any explanation of how low arousal could occur with increases in parasympathetic nervous system activation.

In contrast to the top down tennents of arousal theory, Polyvagal Theory approached the autonomic nervous system as a feedback system that functioned to optimize homeostatic processes of health, growth, and restoration. Through the lens of the Polyvagal Theory, homeostasis has a more nuanced meaning involving the status of feedback circuits involving the bidirectional communication between organs and the brainstem. The traditional autonomic model assumed that homeostasis was relatively stable and was maintained by the competing inputs from the sympathetic and parasympathetic divisions of the autonomic nervous system, although the pathways involved in feedback circuits determining homeostasis were not elaborated. Polyvagal Theory, with its emphasis on bidirectional communication between brain structures and visceral organs, assumes that homeostasis is best described not solely by a static set point, but requires an additional assessment of the systematic perturbations around the set point. Strikingly, within physiology, psychophysiology, and even medicine, there is little acknowledgment of the important role of visceral afferents (a defining feature of a feedback system) that travel primarily through the vagus from several visceral organs to a brainstem center, providing the relevant information to ensure that the output to the organs support homeostatic functions.

Polyvagal Theory required a different quantification strategy, and a new family of metrics (e.g., RSA as an index of vagal regulation of the heart and more recently vagal efficiency) was needed to be developed to accurately monitor the dynamic regulation of the autonomic nervous system. The theory encouraged scientists to look beyond mean levels of variables and to study the periodicities in the physiological signal that represented the features of the feedback system that evolved to optimize homeostasis. Time series methodologies complemented descriptive statistics, and new measures (e.g., RSA) described the systematic perturbations around the set point.

Conceptually, we can visualize the amplitude of RSA as an index of the degree that the autonomic nervous system is supporting either homeostasis or bodily movement, often in support of the mobilization behavioral (i.e., fight/flight) reactions to threat. The vagal brake represents the actions of engaging and disengaging the vagal influences on the heart’s pacemaker. If the vagus no longer influences the heart, heart rate spontaneously increases without any change in sympathetic excitation. The intrinsic heart rate of young healthy adults is about 90 beats per minute. However, baseline heart rate is noticeably slower due to the chronic cardioinhibitory influence of the vagus on the sinoatrial node, the heart’s pacemaker. This chronic cardioinhibitory influence functions as a “vagal brake.” In addition, since the amplitude of RSA represents the strength of the vagal brake, monitoring RSA functionally indexes the homeostatic reserve of the autonomic nervous system to the challenges that we often label as stress. Thus, disruption of homeostasis, indexed by RSA and vagal efficiency, would be an accurate measurable indicator of the impact of challenges and might be a more functional definition of stress than levels of adrenal hormones (e.g., cortisol).

Without sensitive metrics to assess the neural regulation supporting visceral organs, clinical medicine is unable to detect the antecedent disruption in neural regulation that would precede organ damage. Although the sensory pathways of the vagus function as a surveillance system continuously updating brainstem regulatory centers on organ status, this conceptualization of dynamic feedback in the regulation of visceral organs has not been emphasized in the training of physicians. Although end- organ evaluation through biopsy and blood tests dominates the assessment models of visceral organs, tapping into the constant surveillance of visceral organs through vagal or other neural pathways has not been frequently acknowledged by physicians. If you are curious about this statement, just ask your internist what they have learned about the sensory fibers linking the brain with the organs they treat (e.g., heart, kidney, liver, lung).

The introduction of HRV within psychophysiology and especially Polyvagal Theory, required a shift in theoretical orientation regarding heart rate to emphasize that neural influences via feedback produced oscillations in heart rate. In the early studies of HRV, HRV was treated as a descriptive variable without attributing any specific underlying physiological mechanism for the variability of the beat-to-beat variance. HRV was quantified as the variability of beat-to-beat or second-to-second patterns.

HRV cannot be studied without an understanding of the neural regulation of the heart and the construct of a feedback system. However, interest in neural regulation of the heart was not a major concern during the founding years of psychophysiology. Without an understanding of neural mechanisms, the prevalent theoretical perspectives of psychophysiologists in the 1960s was that HRV was error caused by either poor experimental control or inaccurate measurement. When I presented my early studies on HRV, several senior colleagues argued that the findings were spurious and due to inadequate experimental control. Their perspective was consistent with an assumption that heart rate, similar to behavior, required an identifiable ‘external’ stimulus to evoke a reliable response. This perspective highlights the inadequacy of a simplistic stimulus–response (S-R) or cause and effect model. An important tennent of Polyvagal Theory is the role of autonomic state as an intervening variable and placing indices of autonomic state (e.g., RSA, vagal efficiency) as the’O’ in a more explanatory S–O-R model. Our research continues to document the important mediating influence of autonomic state on clinical outcome (see Dale et al., 2022a, 2022b; Kolacz et al., 2020), whether the metric used is autonomic (e.g., RSA, vagal efficiency) or subjective reports of autonomic state (e.g., Cabrera et al., 2017).

In closing, I have been informed by my personal journey studying HRV that certain strategies are beneficial. First, although we are attracted to psychological processes such as cognitions and emotions or mental health diagnoses, we need to respect that higher brain functions are dependent on brainstem foundational survival mechanisms not being triggered into states that support defense. Basically, the higher brain processes are dependent on circuits supporting homeostatic functions (see Porges, 2022). Simiarly, when we focus on medical disorders, we need to acknowledge that the target organs that are expressing these disorders may be directly influenced by these brainstem foundational survival mechanisms and the symptoms may be a direct consequence of this system going into a state that supports defense and not homeostatic functions. Second, ANS states supporting homeostatic functions can be monitored through HRV metrics. However, the sensitivity the HRV metric to neural influences varies by the methodology used. The predictive value of the HRV metric is dependent on its validity to track neural regulation. Specifically, we know most about the neural influences mediating RSA. When carefully quantified, RSA is an excellent index of vagal efferent activity regulated by the ventral vagal nucleus, nucleus ambiguus (Lewis et al., 2012). Third, use of HRV metrics that have neural validity are more accurate indicators of the ‘O’ in S–O-R models and hypothetically should be more sensitive to mental, behavioral, and health. Fourth, since biofeedback as a discipline is focused on optimizing the ‘O’ in the ‘S–O-R’ model, the implementation of more accurate indices of the ‘O’ should result in a more efficient implementation of biofeedback protocols with more optimized outcomes.

## Footnotes

1 Portions of this section ae based on my contributions to the HRV guidelines paper (see Berntson et al., 1997).
